# Anatomical Evaluation of Root and Root Canal Configuration of Permanent Maxillary Dentition in the Population of the Kingdom of Saudi Arabia

**DOI:** 10.1155/2022/3428229

**Published:** 2022-01-15

**Authors:** Mohammed Mashyakhy, Mohammed Awawdeh, Abdulaziz Abu-Melha, Bushra Alotaibi, Nada AlTuwaijri, Nouf Alazzam, Rahaf Almutairi, Reuof Alessa

**Affiliations:** ^1^Department of Restorative Dental Sciences, College of Dentistry, Jazan University, Jazan, Saudi Arabia; ^2^Department of Preventive Dental Sciences, College of Dentistry, King Saud Bin Abdulaziz University for Health Sciences, Riyadh, Saudi Arabia; ^3^Department of Restorative Dental Sciences, College of Dentistry, King Khalid University, Abha, Saudi Arabia; ^4^General Dental Practitioner, Riyadh, Saudi Arabia

## Abstract

**Aim:**

This study is aimed at combining the sample sizes of all studies on permanent maxillary teeth conducted in different regions of the Kingdom of Saudi Arabia (KSA) to obtain a large sample size that represents the population of the KSA. The outcome of these combined studies is compared with international studies in terms of the number of roots, number of canals, and canal configurations on the basis of Vertucci's classification. *Methodology*. The studies were systematically reviewed using the Preferred Reporting Items for Systematic Review and Meta-analysis chart. Studies were included in the analysis if they were conducted in the KSA, involved permanent human maxillary teeth, and had a sample of more than 10 teeth (power). By contrast, studies were excluded if they involved deciduous teeth in the sample size, investigated nonhuman teeth, were not conducted in the KSA, and were case reports, case series, review studies, and anomalies. Relevant literature was searched from PubMed, Scopus, Web of Science, Embase, Cochrane, and Direct Science by two calibrated teams, starting in August 2020, without time limits or language restrictions.

**Results:**

The database searches and cross-referencing identified a total of 19 relevant studies. All maxillary canines (*N* = 1,018) had one root, whereas 98.4% had one canal and 98.3% had Vertucci type I. Moreover, 63.2% of the maxillary first premolars had two roots, and 91.4% had two canals. The most common Vertucci root canal configuration was type IV (64.6%). The maxillary second premolars mostly had one root (84.4%) and one canal (50.4%). The most common canal configuration was Vertucci type I (47.1%). The majority of maxillary first molars had three roots (98.9%), 48.7% of which had three canals, and 46.4% had four canals. The most prevalent feature of the canal morphology of mesiobuccal roots was Vertucci type II (35.3%). The investigated maxillary second molars had three roots, 88.0% of which had three canals.

**Conclusion:**

This systematic review represents the Saudi population since samples were combined from different studies from different regions of the country. Variations in findings were observed in the same group of teeth from different regions and the same region, while the overall combined samples results fell within the range of other international studies.

## 1. Introduction

The main objective of endodontic therapy is to save natural dentition, either by managing or preventing apical periodontitis. Meticulous chemomechanical cleaning, disinfecting, and shaping of the root canal system (RCS), followed by tight-seal obturation, are the most important measures for treating endodontically involved teeth [1]. These steps are particularly important when the pulp of the offending tooth is infected [2] because the inability to reach the whole pulp space or missing main canals leaves tissues and bacteria in RCSs uncleaned and untouched [2–5]. Clinicians face a wide range of RCSs on a daily basis. Comprehensive knowledge of root canal anatomy is paramount to ensure correct diagnosis, successful treatment, and good prognostic outcomes. The intricacy of RCSs involves therapeutic hurdles and obstacles that can jeopardize the fundamental purpose of root canal treatment (RCT) [6, 7]. Since the turn of the 20th century, several in vivo and in vitro approaches, such as root sectioning; canal staining; tooth clearing; microscopic examination [8–10]; two-dimensional radiographic and clinical inspection [11]; three-dimensional technologies, such as cone beam computed tomography (CBCT) [12]; and microcomputed tomography (mCT) [13] have been adopted to investigate the external and internal anatomy of various tooth groups. Consequently, the results of morphological investigations can differ depending on the study technique, population [14], age [15], and gender [16] of the group of interest.

From 2006 to the present, several studies have utilized different methodologies to analyze maxillary permanent dentition anatomy in various populations in the Kingdom of Saudi Arabia (KSA) [11, 13, 17–34]. In some of these studies, the sample was defined as “Saudi population,” whereas in others the sample was described as “Saudi subpopulation” and was from different regions of Saudi Arabia, most of which were from the central region. A critical concern is the representativeness of the samples. Thus, this study searched for studies on groups of teeth conducted in the KSA and systematically reviewed them to obtain a large sample size that represents all regions of the country as a true KSA population sample. These studies were compared with international studies in terms of the number of roots, the number of canals, and root canal configurations, on the basis of Vertucci's classification [10].

## 2. Methodology

### 2.1. Research Question

This review was conducted according to the Preferred Reporting Items for Systematic Review and Meta-analysis (PRISMA) guidelines to answer the research question, “What are the prevalences of the number of roots, number of canals, and root canal configuration of the permanent maxillary teeth?”

### 2.2. Search Strategy

A comprehensive online search of PubMed, Scopus, and Web of Science databases was conducted to identify relevant studies. Additionally, a manual search was performed on the hosting publishers (ScienceDirect, Springer, and Wiley) and individually on the most common endodontic journals (JOE, IJE, AEJ, EEJ, and SEJ) to identify more relevant studies. Different combinations of the following words were used in the search strategy: (“root canal configuration” OR “root canal morphology” OR “root canal anatomy”) AND (“Kingdom of Saudi Arabia” OR “Saudi Arabia” OR “KSA” OR “Saudi”) AND (“maxillary teeth” OR “maxillary”). The last search date was August 18, 2021. Two independent reviewers (N.A. and R.A.) reviewed the extracted studies on the basis of the following inclusion criteria: full-length articles that reported some or all study variables (number of roots, number of canals, or Vertucci's classification system), conducted on Saudi subjects (in vivo) or teeth extracted from Saudi subjects (in vitro), and published in English. No time limit was selected for the search. All irrelevant studies, including abstracts, editorials, case reports, reviews, and studies with mixed populations, were excluded from the analysis. In the first round of review, the studies initially extracted were reviewed on the basis of their titles and abstracts, and irrelevant studies were excluded. The full text of the remaining studies was then reviewed for inclusion in the second round of review. Moreover, the bibliography lists of the full texts of the included studies were screened for any possible relevant studies not included in the first search. Any disagreement was discussed with a third reviewer who was a specialist in endodontics (M.M.) until the team reached a consensus.

### 2.3. Data Extraction

The following parameters were considered in the evaluation of the studies: authors (first author), year, region, design of the study and research tool, investigated variables, number, gender, and age of recruited subjects, type of teeth, and the number of teeth. The main outcomes included the number of roots, the number of canals, and canal morphology according to Vertucci's classification. The secondary outcomes included the presence of additional canals (e.g., MB2 or MB3). The data were extracted to a spreadsheet (MS Excel) and tabulated according to the type of teeth. The frequency and percentage of each variable were reported, including the total of each category.

## 3. Results

### 3.1. Study Selection

**Figure 1:**
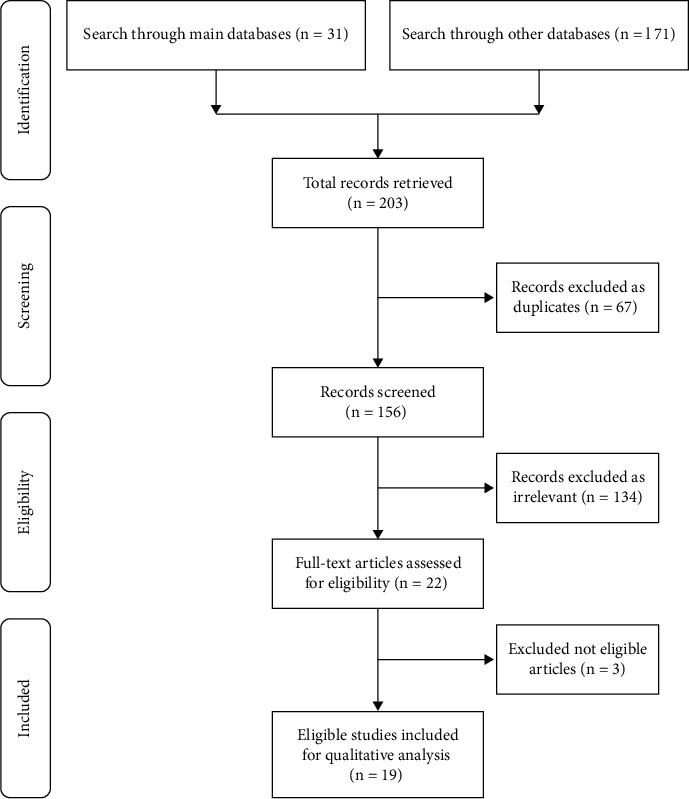
Flowchart of methodology according to PRISMA guidelines.

A total of 203 studies were retrieved from the database search. In the first round of review, 67 studies were removed as duplicates, and 134 studies were excluded as irrelevant according to their titles and abstracts. The full texts of the remaining 22 studies were reviewed in the second round of review for eligibility. Finally, 19 studies were included in the qualitative analysis (Figure 1).

### 3.2. Characteristics of the Included Studies

A total of 14 studies were conducted on Saudi subjects (in vivo), whereas five studies were conducted on the extracted teeth (in vitro) of Saudi subjects. For radiological investigation, 14 studies used CBCT, two studies utilized mCT, and three studies utilized periapical X-ray (PA). In terms of the distribution of the studies, eight, three, three, three, and two studies were conducted in the central, northern, western, southern, and eastern regions of the KSA, respectively. A total of 3,981 subjects were involved in these studies (seven studies did not report the number of subjects). The age of the subjects ranged from 18 years to 75 years (10 studies did not report the age of the participants). With regard to gender distribution, 1,709 were males, and 2,028 were females (nine studies did not report the gender distribution). The external and internal anatomy and morphology of 7,404 teeth were investigated by these studies. However, no study investigated the maxillary central and lateral incisors. Two studies investigated maxillary canines (*N* = 1,018 teeth), eight studies assessed maxillary first premolars (*N* = 2,314 teeth), seven studies evaluated maxillary second premolars (*N* = 2,018 teeth), nine studies examined maxillary first molars (*N* = 1,662 teeth), and three studies focused on maxillary second molars (*N* = 392 teeth). With regard to the variables of interest, eight studies reported the number of roots, number of canals, and used Vertucci's classification system; two studies described the number of roots and number of canals; two studies reported the number of roots and Vertucci's classification system; and one study described the number of canals only. However, six studies investigated the additional canals of the mesiobuccal roots of the maxillary first and second molars. More details are presented in Table 1.

### 3.3. Main Outcome Measures

#### 3.3.1. Maxillary Canines

As shown in Table 2, all the investigated canine teeth (*N* = 1,018 teeth) had one root, of which 98.4% (*N* = 1,002 teeth) had one canal, and 1.6% (*N* = 16 teeth) had two canals. In total, 98.3% (*N* = 1,001 teeth) had Vertucci type I, and 0.7% (*N* = 7 teeth) had Vertucci type III. Only one study reported Vertucci type II (*N* = 3 teeth) and type V (*N* = 7 teeth).

#### 3.3.2. Maxillary First Premolars

Seven studies investigated the number of roots (*N* = 1,851 teeth), of which 63.2% (*N* = 1,170 teeth) had two roots, 35.5% (*N* = 657 teeth) had one root, and 1.3% (*N* = 24 teeth) had three roots. Among six studies that investigated the number of canals (*N* = 1,860 teeth), 91.4% (*N* = 1,700 teeth) had two canals. However, 6.0% had one canal (*N* = 124 teeth), 1.8% had three canals (*N* = 34 teeth), and only one study reported two teeth with four canals. Five studies investigated the canal morphology of 1,495 teeth. Among these studies, 16.3%, 64.6%, and 6.8% were Vertucci types II, IV, and V, respectively. However, four studies reported Vertucci types I (5.8%) or III (3.4%), three studies reported Vertucci types VI (0.7%) or VIII (1.3), two studies reported Vertucci type VII (0.4%), and only one study reported other canal configurations (0.7%). More details are presented in Table 3.

#### 3.3.3. Maxillary Second Premolars

Six studies investigated for the number of roots of 1,587 maxillary second premolars. Maxillary second premolars with one root were the most prevalent (84.4%), followed by maxillary second premolars with two roots (15.0%). Only three studies reported maxillary second premolars with three roots (0.6%). Five studies investigated the number of canals of 1,590 teeth. Teeth with one canal were the most prevalent (50.4%), followed by teeth with two canals (48.6%). Three studies reported teeth with three canals (1.0%), and no study reported teeth with four canals. Five studies investigated the root canal morphology of 1,387 teeth. All of these studies reported Vertucci type I (47.1%), II (16.0%), III (9.1%), IV (15.8%), and V (8.0%). Four studies reported Vertucci type VII (1.2%), and two studies reported Vertucci type VI (0.7%), VIII (0.6%), or other canal configurations (1.6%). More details on the studies and percentages are provided in Table 3.

#### 3.3.4. Maxillary First Molars

Only three studies investigated the number of roots of maxillary first molars (*N* = 651 teeth). Most teeth had three roots (98.9%). Only one study reported one tooth (0.2%) with two roots, and another study reported six teeth (0.9%) with four roots. These studies also investigated the number of canals. About half of the samples (48.7%) had three canals, and only one study reported 13 (2.0%) teeth with two canals (Table 4). Only two studies investigated the internal canal morphology of all roots, and one study examined the internal canal morphology of mesiobuccal root only. The most prevalent feature of the canal morphology of mesiobuccal roots was Vertucci type II (35.3%), followed by type I (27.1%). For distobuccal roots, 99.3% (*N* = 427 teeth) had Vertucci type I, 0.3% (*N* = 1 tooth) had Vertucci type III, and 0.3% (*N* = 1 tooth) had Vertucci type V. However, all palatal roots (*N* = 430 teeth) had Vertucci type I (Table 5).

#### 3.3.5. Maxillary Second Molars

One study examined the number of roots and canals of 200 maxillary second molars. All maxillary second molars had three roots, 88.0% of which (*N* = 176 teeth) had three canals (Table 4). However, this study did not report the Vertucci classification system.

### 3.4. Secondary Outcome Measures

Nine studies (*N* = 1662 teeth) explored the additional canals in maxillary first molars, particularly in the mesiobuccal roots. All studies reported one additional mesiobuccal canal (MB2) with a prevalence of 46.4% (*N* = 771 teeth). Only two studies found a second additional mesiobuccal canal (MB3) in seven teeth (0.4%). For maxillary second molars, three studies (*N* = 392 teeth) reported one additional mesiobuccal canal (MB2) in 80 teeth (20.4%), whereas only one study reported the second additional mesiobuccal canal (MB3) in four teeth (1.0%). More details are given in Table 4.

## 4. Discussion

Root canal anatomy may impose a clinical burden on dentists. Overcoming these difficulties is one of the most relevant challenges that may emerge during endodontic procedures. Potential complications during RCT can be anticipated with a comprehensive understanding and knowledge of RCS in each group of teeth. However, the internal and external morphologies of teeth may vary according to age [35, 36], ethnicity [14, 37, 38], gender [16, 39–41], and geographic region [42]. These differences may explain the stark differences in tooth anatomy within the same or different regions, similar to those found in our study.

The effect of different methodologies in assessing the root canal anatomy is well known, since the mCT systems can achieve a micron resolution that nearly match with histology. In addition, the degree of accuracy 3D technology like CBCT and mCT offers is uncompared to conventional radiography and/or clinical observation [9, 11, 13, 35]. So, regardless of the methodologies used, in this systematic review, we collected all studies on permanent maxillary dentition in various Saudi populations to obtain a large sample size of a given group of teeth from different regions of the country.

### 4.1. Maxillary Canines

No studies investigated anterior teeth, except for two studies that evaluated maxillary canines [17, 22], which showed that the anterior teeth had one root (100%), 98.4% had one canal, and 1.6% had two canals. Vertucci type I was the most predominant canal configuration (98.3%).

Our results were consistent with those of a study conducted in Malaysia, which reported that maxillary canines had only one root and could be assigned to Vertucci type I [43]. Another study conducted in Portugal showed that all teeth had only one root, and only 1.4% had two canals [44].

### 4.2. Maxillary First Premolars

The presence of two roots in maxillary first premolars was predominant (63.2%), followed by one root (35.5%), and three roots (1.3%). Most of the teeth had two canals (91.4%), with Vertucci type IV (64.6%) as the most prevalent. By comparison, other studies on different populations that used different methodologies reported that the prevalence of maxillary first premolars with two roots range from 33% to 84%, and those with one root range from 22% to 66%. Finally, those with three roots ranged from 0% to 6% [45–50]. Our study fell within the higher range with regard to maxillary first premolars with two roots, and within the lower range with regard to maxillary first premolars with one root.

A systematic review [51] investigated the internal morphology of maxillary first premolars. It included 41 studies that used different techniques with a total of 10,013 teeth. It reported that 86.6% of the teeth had two canals, 11.2% had one canal, and only 2.2% had three canals. Vertucci type IV canal configuration was the most prevalent (64.8%). The results of this review were very close to our findings.

### 4.3. Maxillary Second Premolars

In this study, maxillary second premolars with one root were the most prevalent (84.4%), followed by those with two roots (15.0%). Maxillary second premolars with one canal were the most prevalent (50.4%), followed by those with two canals (48.6%). Vertucci type I (47.1%) was the predominant type.

Similarly, other studies of different populations reported that approximately 67% to 94.4% of maxillary second premolars had a higher prevalence of one root, about 50% of which had either one or two root canals [44, 52–57]. Maxillary second premolars have a widely different internal morphology, which poses a challenge to practitioners during RCT [58–61]. When the maxillary second premolars have two canals, all Vertucci types, lateral canals, and anastomoses can be expected [60]. Our results observed all canal types and extra canal configurations.

### 4.4. Maxillary First Molars

Corbella et al. [62] reviewed the studies that examined the root canal morphology of maxillary first molars. They found that 96.2% of maxillary first molars had three roots, and root fusion occurred approximately 5.2% of the time when the teeth had two or more roots. Our study found that 98.9% of the maxillary first molars had three roots. However, root fusion was not evaluated in this study. A previous study of a Saudi subpopulation reported that the prevalence of fused-rooted maxillary first molars was 7% [6].

Out of 8,399 maxillary first molars, 56.8% of MB roots had two or more canals, whereas 43.1% had one canal. The incidence of MB2 ranged from 25% to 96% [63]. Moreover, 46.4% of the maxillary first molars had four canals, and 48.7% had three canals. The most prevalent feature of canal morphology of mesiobuccal roots with two canals was Vertucci type II (35.3%). A previous study that utilized CBCT reported that the average percentage of maxillary first molars with an additional canal in MB root was 59.3% [62], which was higher than that observed in this study. Moreover, the prevalence of this condition was higher in a Korean population (73.3%) [39, 41] than that in the present study. Our study reported a lower prevalence of type I (27.1%).

Type I, II, and IV canal configurations are reportedly the most common internal morphology of MB roots in different populations (42% to 75.1% had type I) [64–68]. By contrast, our study observed a lower prevalence of type I (27.1%).

### 4.5. Maxillary Second Molars

Many studies reported that the prevalence of maxillary second molars with three roots is higher than those with four roots [41, 65, 69–72], consistent with our findings where all samples had three roots. Few studies [41, 69, 72–74] have evaluated root fusion in maxillary second molars. A study in Brazil showed that the prevalence of root fusion in maxillary second molars was high (7.94%). However, fused-rooted teeth were not included in this study. Mashyakhy et al. [6] reported that the incidence of root fusion and internal canal morphology of fused-rooted maxillary second molars was high (21%). The presence of second mesiobuccal canal reportedly ranged from 11.53% to 93.7% [75], with type II as the predominant canal configuration. Our findings fell within the lower range (about 20%). No study has evaluated internal canal configurations.

With regard to secondary outcomes, only studies that focused on the presence of other canal/canals in the MB root of maxillary first and second molars were analyzed. Results showed that MB2 was more prevalent in maxillary first molars than in maxillary second molars.

Out of 1,662 maxillary first molars, the prevalence of MB2 was 46.4%. Only two studies found that the presence of MB3 was rare (0.4%). With regard to maxillary second molars, three studies (*N* = 392 teeth) reported that the prevalence of MB2 was 20.4%. One study reported that the prevalence of MB3 was 1.0% (*N* = 4 teeth). A global CBCT study reported that the prevalence of a second canal in MB roots was 73.8% (48% to 97.6%) [76]. Our results fell within the lower range of this result. On the basis of their analysis of samples from 24 countries worldwide that covered 41 population groups with a wide variety among different populations, Martins et al. [77] reported that the average prevalence of MB2 in the first and second molars was 69.6% and 39.0%, respectively. These figures were higher than our findings for both maxillary teeth.

Our study observed that the studies analyzed herein had wide differences among the same population from different regions. The differences were notable regardless of whether the same and/or a different methodology was used in examining the same group of teeth, particularly in the analysis of the number of canals and canal configurations.

Previous studies examined root canal morphology via different methodologies, including tooth clearing and staining [10, 54, 78] and mCT [79], which can provide a highly accurate and precise description of RCS. Although these methodologies can give a clear picture of the internal morphology of a root, they can be done on extracted teeth only. CBCT is a three-dimensional radiography technique. It is modified canal staining and clearing that can be used to detect root canal anatomy accurately [80]. CBCT is a widely available noninvasive in vivo methodology for addressing RCS; it can overcome the limitations of two-dimensional intraoral radiography [81]. The studies included herein involved different techniques from different regions. Thus, they reported different results. Nevertheless, they collectively provided an invaluable insight into the root canal anatomy of permanent dentition in the entire Saudi population.

Unfortunately, detailed epidemiological data cannot be obtained from most laboratory studies because some variables are unknown or impossible to acquire. Thus, in most cases, evaluation is performed using small sample sizes. Consequently, an observational study using CBCT imaging is the best approach for estimating the frequency of individuals with specific root/canal morphologies. It allows the analysis of full dentition of several patients collected from a specific population in a consecutive manner. Owing to the widespread use of CBCT technology, several studies on root and root canal anatomy from different countries have been conducted.

### 4.6. Limitations

The 19 studies from the different regions of KSA included herein utilized different methodologies. Thus, demographic data were not obtained to evaluate the effects of gender and age on the present findings. Moreover, the studies were not separated according to methodologies or classified as in vivo or in vitro because the number of studies of different groups of teeth was small. CBCT could be the best favourable way to study dental anatomy, since it is an in vivo noninvasive technology where one scan can include all permanent dentition with high quality, and all the demographic data can be evaluated and compared for better outcome [40, 43, 44]. Further multicenter studies from all regions of the country should utilize in vivo CBCT methodology to obtain a large sample size that represents the entire Saudi population, with more detailed information on the effect of age and gender.

## 5. Conclusion

Regardless of the methodology, the anatomical studies included in the present report vary between different regions of the same country, though they share the same ethnicity. Thus, root canal morphology must be carefully evaluated to ensure successful endodontic treatment. A CBCT with a small field of view should be considered when intraoral periapical radiography is inconclusive to understand the patient's tooth anatomy and achieve a successful outcome.

## Figures and Tables

**Table 1:** General characteristics of the included studies.

Study	Year	Region	Method	Investigation	No. subjects	Gender	Age	Type of teeth	No. of teeth
Agwan et al.	2015	Northern	In vivo; PA	MB roots	100	53M, 47F	29 ± 3	First molars	100
Alfouzan et al.	2019	Central	In vitro; micro-CT	MB roots	NR	NR	NR	First molars	35
								Second molars	30
Al-Fouzan et al.	2013	Central	In vivo; PA	MB roots	NR	NR	NR	First molars	308
								Second molars	162
Al-Habib et al.	2021	Western	In vivo; CBCT	MB roots	106	44M, 62F	20–65	First molars	106
Almohaimede et al.	2021	Central	In vivo; CBCT	#roots; #canals; Vertucci	1328	565M, 763F	18–74	Canines	634
Al-Nazhan et al.	2012	Central	In vivo; CBCT	#canals	628	268M, 360F	NR	First premolars	463
								Second premolars	431
Al-Nazhan et al.	2005	Central	In vivo; PA	MB roots	332	171M, 181F	NR	First molars	352
Alqedairi et al.	2018	Central	In vivo; CBCT	#roots; Vertucci	NR	NR	16–71	First premolars	334
								Second premolars	318
Alrahabi et al.	2015	Western	In vitro; CBCT	#roots; #canals; Vertucci	NR	NR	20–60	First molars	100
Al-Shehri et al.	2017	Central	In vivo; CBCT	#roots; #canals; Vertucci	207	103M, 104F	16–75	First molars	351
Al-Swilem et al.	2018	Northern	In vivo; CBCT	MB roots	110	NR	NR	First molars	110
Al-Zubaidi et al.	2021	Northern	In vivo; CBCT	#roots; #canals; Vertucci	500	250M, 250F	18–60	First premolars	500
								Second premolars	500
Atieh et al.	2008	Eastern	In vitro; CBCT	#roots; #canals	NR	NR	NR	First premolars	246
Elhejazi et al.	2021	Central	In vivo; CBCT	#roots; #canals	100	55M, 45F	NR	First premolars	200
								Second premolars	200
								First molars	200
								Second molars	200
Elkady et al.	2013	Western	In vivo; CBCT	#roots; Vertucci	64	NR	NR	First premolars	120
								Second premolars	110
Elnour et al.	2016	Eastern	In vitro; micro-CT	#roots; #canals; Vertucci	NR	NR	NR	Second premolars	100
Maghfuri et al.	2019	Southern	In vitro; CBCT	#roots; #canals; Vertucci	NR	NR	NR	First molars	100
Mashyakhy et al.	2019	Southern	In vivo; CBCT	#roots; #canals; Vertucci	208	100M, 108F	17–62	Canines	384
Mashyakhy et al.	2021	Southern	In vivo; CBCT	#roots; #canals; Vertucci	208	100M, 108F	17–59	First premolars	351
								Second premolars	359

NR: not reported.

**Table 2:** Number of roots, number of canals, and root canal configuration of maxillary canines among Saudi populations.

Study (year)	Region	Method	Sample	# roots (%)			# canals (%)			Vertucci's system (%)							
				1	2	1	2	I	II	III	IV	V	VI	VII	VIII	Others
Almohaimede et al. [22]	Central	CBCT	634	634		622	12	621	3	3		7				
		In vivo		(100.0)		(98.1)	(1.9)	(97.9)	(0.5)	(0.5)		(1.1)				

Mashyakhy et al. [6]	Southern	CBCT	384	384		380	4	380		4						
		In vivo		(100.0)		(99.0)	(1.0)	(99.0)		(1.0)						

Total			1018	1018		1002	16	1001	3	7		7				
				(100.0)		(98.4)	(1.6)	(98.3)	(0.3)	(0.7)		(0.7)				

**Table 3:** Number of roots, number of canals, and root canal configuration of maxillary first and second premolars among Saudi populations.

Study (year)	Region	Method	Sample	# roots (%)			# canals (%)				Vertucci's system (%)								
				1	2	3	1	2	3	4	I	II	III	IV	V	VI	VII	VIII	Others
First premolars																			
Mashyakhy et al. [14]	Southern	CBCT	351	143	202	6	13	327	9	2	13	24	27	224	52	1			10
		In vivo		(40.7)	(57.5)	(1.8)	(3.7)	(93.1)	(2.6)	(0.6)	(3.7)	(6.8)	(7.7)	(63.8)	(14.8)	(0.3)			(2.8)
Al-Zubaidi et al. [27]	Northern	CBCT	500	199	293	8	39	453	8		26	164	3	289	10			8	
		In vivo		(39.8)	(58.6)	(1.60)	(7.8)	(90.6)	(1.6)		(5.2)	(32.8)	(0.6)	(57.8)	(2)			(1.6)	
Elhejazi et al. [29]	Central	CBCT	200	122	78		33	167											
		In vivo		(61.0)	(39.0)		(16.5)	(83.5)											
Maghfuri et al. [30]	Southern	CBCT	100	36	61	3		97	3			7		75	13	2		3	
		In vitro		(36.0)	(61.0)	(3.0)		(97.0)	(3.0)			(7.0)		(75.0)	(13.0)	(2.0)		(3.0)	
Alqedairi et al. [28]	Central	CBCT	334	79	251	4					36	28	6	236	13	7	1	7	
		In vivo		(23.7)	(75.1)	(1.2)					(10.8)	(8.4)	(1.8)	(70.6)	(3.9)	(2.1)	(0.3)	(2.1)	
Elkady et al. (2013)	Western	CBCT	120	34	86						6	6	12	84	8		4		
		In vivo		(28.3)	(71.7)						(5.0)	(5.0)	(1.0)	(70.0)	(6.7)		(3.3)		
Al-Nazhan et al. [26]	Central	PA	463				17	435	11										
		In vivo					(3.7)	(94.0)	(2.3)										
Atieh et al. (2008)	Eastern	PA	246	44	199	3	22	221	3										
		In vitro		(17.9)	(80.9)	(1.2)	(8.9)	(89.9)	(1.2)										
Total			2314	657	1170	24	124	1700	34	2	81	229	48	908	96	10	5	18	10
				(35.5)*^α^*	(63.2)*^α^*	(1.3)*^α^*	(6.0)*^β^*	(91.4)*^β^*	(1.8)*^β^*	(0.1)*^β^*	(5.8)*^δ^*	(16.3)*^δ^*	(3.4)*^δ^*	(64.6)*^δ^*	(6.8)*^δ^*	(0.7)*^δ^*	(0.4)*^δ^*	(1.3)*^δ^*	(0.7)*^δ^*
Second premolars																			
Mashyakhy et al. [14]	Southern	CBCT	359	316	43		137	219	3		137	39	55	69	44	4	8		3
		In vivo		(88.0)	(12.0)		(38.2)	(61.0)	(0.8)		(38.2)	(10.9)	(15.3)	(19.2)	(12.3)	(1.1)	(2.2)		(0.8)
Al-Zubaidi et al. [27]	Northern	CBCT	500	416	79	5	348	147	5		302	82	32	64	14		1	5	
		In vivo		(83.2)	(15.8)	(1.0)	(69.6)	(29.4)	(1.0)		(60.4)	(16.4)	(6.4)	(12.8)	(2.8)		(0.2)	(1.0)	
Elhejazi et al. [29]	Central	CBCT	200	186	14		115	85											
		In vivo		(93.0)	(7.0)		(57.5)	(42.5)											
Alqedairi et al. [28]	Central	CBCT	318	271	46	1					157	82	16	37	18	5		3	
		In vivo		(85.2)	(14.5)	(0.3)					(49.4)	(25.8)	(5.0)	(11.6)	(5.7)	(1.6)		(0.9)	
Elnour et al. (2016)	Eastern	CT	100	67	30	3	30	65	5		17	7	9	23	23		2		19
		In vitro		(67.0)	(30.0)	(3.0)	(30.0)	(65.0)	(5.0)		(17.0)	(7.0)	(9.0)	(23.0)	(23.0)		(2.0)		(19.0)
Elkady et al. (2013)	Western	CBCT	110	84	26						40	12	14	26	12		6		
		In vivo		(76.4)	(23.6)						(36.3)	(10.9)	(12.7)	(23.6)	(10.9)		(5.4)		
Al-Nazhan [26]	Central	PA	431				171	256	4										
		In vivo					(39.7)	(59.4)	(0.9)										
Total			2018	1340	238	9	801	772	17		653	222	126	219	111	9	17	8	22
				(84.4)*^η^*	(15.0)*^η^*	(0.6)*^η^*	(50.4)*^λ^*	(48.6)*^λ^*	(1.0)*^λ^*		(47.1)*^μ^*	(16.0)*^μ^*	(9.1)*^μ^*	(15.8)*^μ^*	(8.0)*^μ^*	(0.7)*^μ^*	(1.2)*^μ^*	(0.6)*^μ^*	(1.6)*^μ^*

*^α^* The percentage is out of 1,851 teeth (studies that investigated the number of roots of 1^st^ premolars); *^β^*the percentage is out of 1,860 teeth (studies that investigated the number of canals of 1^st^ premolars); *^δ^*the percentage is out of 1,405 teeth (studies that investigated the Vertucci system of 1^st^ premolars); *^η^*the percentage is out of 1,587 teeth (studies that investigated the number of roots of 2^nd^ premolars); *^λ^*the percentage is out of 1,590 teeth (studies that investigated the number of canals of 2^nd^ premolars); *^μ^*the percentage is out of 1,387 teeth (studies that investigated the Vertucci system of 2^nd^ premolars).

**Table 4:** Number of roots and number of canals of maxillary first and second molars among Saudi populations.

Study (year)	Region	Method	Sample	# roots (%)				# canals (%)				
				1	2	3	4	1	2	3	MB2	MB3
Maxillary first molars												
Al-Habib et al. (2021)	Western	CBCT	106								92	
		In vivo									(86.8)	
Alhejazi et al. (2021)	Central	CBCT	200			200				146	54	
		In vivo				(100.0)				(73.0)	(27.0)	
Alfouzan et al. [21]	Central	CT	35								28	6
		In vitro									(80.0)	(17.1)
Alswilem et al. [24]	Northern	CBCT	110								46	
		In vivo									(41.8)	
Al-Shehri et al. [33]	Central	CBCT	351		1	350			13	142	195	1
		In vivo			(0.3)	(99.7)			(3.7)	(40.4)	(55.6)	(0.3)
Alrahabi et al. (2015)	Western	CBCT	100			94	6			29	71	
		In vitro				(94.0)	(6.0)			(29.0)	(71.0)	
Agwan et al. (2015)	Northern	PA	100								45	
		In vivo									(45.0)	
Al-Fouzan et al. [18]	Central	PA	308								158	
		In vivo									(51.3)	
Al-Nazhan et al. (2005)	Central	PA	352								82	
		In vivo									(23.3)	
Total			1662		1	644	6		13	317	771	7
					(0.2)*^α^*	(98.9)*^α^*	(0.9)*^α^*		(2.0)*^β^*	(48.7)*^β^*	(46.4)*^η^*	(0.4)*^η^*
Maxillary second molars												
Alhejazi et al. (2021)	Central	CBCT	200			200				176	24	
		In vivo				(100.0)				(88.0)	(12.0)	
Alfouzan et al. [21]	Central	CT	30								24	4
		In vitro									(80.0)	(13.3)
Al-Fouzan et al. (20[61]13)	Central	PA	162								32	
		In vivo									(19.7)	
Total			392			200				176	80	4
						(100.0)*^δ^*				(88.0)*^λ^*	(20.4)*^μ^*	(1.0)*^μ^*

*^α^* The percentage is out of 651 teeth (studies that investigated the number of roots of 1^st^ molars); *^β^*the percentage is out of 651 teeth (studies that investigated the number of canals of 1^st^ molars); *^η^*the percentage is out of 1,662 teeth (studies that investigated the number of canals of MB roots of 1^st^ molars); *^δ^*the percentage is out of 200 teeth (studies that investigated the number of roots of 2^nd^ molars); *^λ^*the percentage is out of 200 teeth (studies that investigated the number of canals of 2^nd^ molars); *^μ^*the percentage is out of 392 teeth (studies that investigated the number of canals of MB roots of 2^nd^ molars).

**Table 5:** Root canal configuration of maxillary first molars among Saudi populations.

Study (year)	Region	Method	Sample	Type of root	Vertucci's system (%)								
					I	II	III	IV	V	VI	VII	VIII	Others
Al-Habib et al. (2021)	Western	CBCT	106	Mesiobuccal		62		44					
				Roots		(58.5)		(41.5)					
				Distobuccal									
		In vivo		Roots									
				Palatal									
				Roots									

Al-Shehri et al. [33]	Central	CBCT	330	Mesiobuccal	116	80	12	110	6	4		1	1
				Roots	(35.1)	(24.4)	(3.6)	(33.3)	(1.8)	(1.2)		(0.3)	(0.3)
				Distobuccal roots	327		1		1				1
		In vivo			(99.0)		(0.4)		(0.3)				(0.3)
				Palatal	330								
				Roots	(100.0)								

Alrahabi et al. (2015)	Western	CBCT	100	Mesiobuccal	29	47	12	12					
				Roots	(29.0)	(47.0)	(12.0)	(12.0)					
				Distobuccal roots	100								
		In vitro			(100.0)								
				Palatal	100								
				Roots	(100.0)								

Total			536	Mesiobuccal	145	189	24	166	6	4		1	1
				Roots	(27.1)*^α^*	(35.3)*^α^*	(4.5)*^α^*	(31.0)*^α^*	(1.1)*^α^*	(0.7)*^α^*		(0.2)*^α^*	(0.2)*^α^*
				Distobuccal	427		1		1				
				Roots	(99.3)*^β^*		(0.3)*^β^*		(0.3)*^β^*				
				Palatal	430								
				Roots	(100.0)*^η^*								

*^α^* The percentage is out of 536 teeth (studies that investigated the Vertucci's system of MB roots of 1^st^ molars); *^β^*the percentage is out of 430 teeth (studies that investigated the Vertucci's system of DB roots of 1^st^ molars); *^η^*the percentage is out of 430 teeth (studies that investigated the Vertucci's system of *P* roots of 1^st^ molars).

## Data Availability

The data supporting the findings of this review are already included.
